# An annotated checklist of Conopidae (Diptera) of Armenia

**DOI:** 10.3897/zookeys.1286.187591

**Published:** 2026-07-23

**Authors:** Nonna Grigoryan, Matthieu Aubert, Gayane Karagyan, Mark Kalashian, Tigran Ghrejyan

**Affiliations:** 1 Scientific Centre of Zoology and Hydroecology of National Academy of Sciences of Republic of Armenia, P. Sevak, 7, Yerevan, 0014, Armenia Scientific Centre of Zoology and Hydroecology of National Academy of Sciences of Republic of Armenia Yerevan Armenia; 2 Observatoire des Abeilles, 4 chemin de la Foux, 34380 Pegairolles-de-Bueges, France Observatoire des Abeilles Pegairolles-de-Bueges France

**Keywords:** Faunistics, habitat preferences, Myopa, new country records, Thecophora, thick-headed flies

## Abstract

This study presents the first comprehensive, country-level checklist of Conopidae (Diptera) from Armenia, based on material collected during 2024–2025 expeditions and specimens deposited in the collections of the Scientific Center of Zoology and Hydroecology (Yerevan). A total of 32 species from 12 genera are recorded, including six species newly reported for Armenia: *Physocephala antiqua* (Wiedemann, 1830), *Myopa buccata* (Linnaeus, 1758), *Myopa picta* Panzer, 1797, *Myopa variegata* Meigen, 1804, *Thecophora atra* (Fabricius, 1775), and *Thecophora cinerascens* (Meigen, 1804). Species’ distributions show clear habitat associations along an elevational gradient, with most taxa occurring in mid-elevation mountain steppes and grasslands (≈ 800–2000 m), while alpine and subalpine meadows (≈ 2000–2600 m) support several high-elevation species. These results identify Armenia as an important area for conopid diversity in the Transcaucasia.

## Introduction

The family Conopidae (Diptera), commonly known as thick-headed flies, comprises acalyptrate Diptera generally characterised by a swollen head and, in Conopinae, an elongate proboscis and a wasp-like appearance, with females in several genera possessing a modified curved abdomen adapted for oviposition. Members of this family are obligate endoparasitoids of adult Aculeata (Hymenoptera), particularly bees (from the families Apidae, Andrenidae, Halictidae) and wasps (Vespidae), and at least few species play a notable ecological role in regulating pollinator populations (Smith 1969; Schmid-Hempel and Müller 1991; Smit et al. 2018).

Globally, the family Conopidae comprises over 800 described species in approximately 57 genera, with the highest diversity occurring in the Palearctic and Nearctic regions (Stuke 2017). Adults are frequently observed visiting flowering patches, which serve both as nectar sources and as sites where female conopid flies intercept foraging hymenopteran hosts and insert eggs into their metasoma. The larvae develop endoparasitically (Smith 1969; Stuke 2017).

Despite their ecological importance and distinctive biology, the fauna of Conopidae in Armenia remains poorly studied, whereas the family has been more extensively investigated in neighbouring countries, particularly Turkey and Iran (Stuke et al. 2012; Koçak and Kemal 2013; Khaghaninia and Kazerani 2014a, 2014b). Armenia, located at the intersection of several biogeographical regions, supports a rich and diverse insect fauna (Kalashian et al. 2023), highlighting the need for focused studies on poorly known dipteran families such as Conopidae. Available information on Armenian Conopidae has primarily originated from older Soviet-era studies, which frequently lack detailed locality data and/or ecological context (Paramonov 1927; Zimina 1960, 1963a, 1963b, 1970, 1975, 1976). These data were generalised in the Catalogue of Palearctic Diptera (Chvála and Smith 1988) and simply repeated in Arzumanyan et al. (2025) who listed from Armenia 18 species without any additional information.

Given the increasing interest in pollinator–parasitoid interactions and biodiversity conservation, updated knowledge of conopid diversity and distribution is essential. The present study addresses this gap by documenting the species composition of Conopidae in Armenia and providing verified records and distributional data, thereby establishing a baseline for future ecological, taxonomic, and conservation-oriented research in the region.

## Material and methods

The study is based primarily on material collected by the authors and their collaborators during systematic field investigations conducted throughout Armenia in 2024–2025, encompassing all major geographic regions and habitat types covering a broad altitudinal range (approximately 400–2800 m a.s.l.), including semi-desert landscapes, steppe grasslands, forest edges, and montane meadow habitats. Adult flies were collected using standard entomological sweep nets and immediately transferred to killing jars charged with ethyl acetate. Sampling was carried out primarily during sunny weather conditions, when adult conopids exhibit peak activity and frequently visit flowering plants. Collected specimens were air-dried, pinned, and labeled following standard entomological procedures. In addition to newly collected specimens, historical material from the conopid collection of the Scientific Center of Zoology and Hydroecology (Yerevan), was also examined. Photographs were taken with a Canon EOS 800D digital camera, equipped with a Canon MP-E65 mm f/2.8 and LAOWA 100 mm f/2.8 Ultra Macro APO lenses, and mounted on a Stack Shot Macro Rail package (Cognisys Inc.). Photo stacking was performed using Helicon Focus Pro software.

Species identification was performed based on morphological characters examined under a stereomicroscope (Zeiss Stemi 305). For generic and species-level identification, the following keys were used: Smith (1969); Stuke (2007) for *Dalmannia* Robineau-Desvoidy, 1830; De Bree et al. (2014) for *Leopoldius* Rondani, 1843; Kröber (1916), Zimina (1963a), and Stuke and Clements (2008) for *Myopa* Fabricius, 1775; Stuke (2014b, 2016) for *Physocephala* Schiner, 1861; Zimina (1963b) and Bessonova and Galinskaya (2016) for *Sicus* Scopoli, 1763; Kahanpää (2007) and Stuke (2006) for *Thecophora* Rondani, 1845; De Bree and Smit (2012), and Mei and Stuke (2008) for *Zodion* Latreille, 1796.

All examined material is deposited in the Scientific Center of Zoology and Hydroecology, National Academy of Sciences of the Republic of Armenia. Label data are provided in English, with locality names standardised to current toponyms following widely used geospatial resources such as Google Earth, Google Maps, and GeoNames. Historical place names were interpreted using the Dictionary of Armenian Toponyms (Hakobyan et al. 2001). Examined material is arranged according to the present administrative divisions of the Republic of Armenia (marzes, hereafter referred to as provinces), with Yerevan treated as a separate administrative unit; province names are shown in bold in order from northwest to southeast (see Map, Fig. 17). Geographic coordinates were recorded using handheld GPS units. Data on the occurrence of conopid flies in the main types of habitats are provided. Species newly recorded for Armenia are indicated by an asterisk (*). Species previously reported only from broader regions (e.g. “Transcaucasia”) are not marked with an asterisk but are treated as first explicit records for Armenia.

Higher-level classification follows the phylogenetic framework of Gibson and Skevington (2013), while the internal arrangement and nomenclature of the subfamilies follow the morphological concepts of Stuke (2014a). Global distributional records were provided generalizing data from *The Catalogue of Palaearctic Diptera* (Chvála and Smith 1988) and *World Catalogue of Insects* (Stuke 2017); the latter work was consulted specifically to verify historical synonymies and legacy distribution records within the Transcaucasian region.

To investigate the geometric constraints on species richness, the mid-domain effect (MDE) was analyzed by defining the elevation gradient as a bounded domain (480–2630). The domain was divided into equal intervals to assess the overlap of species ranges. Species richness was calculated as the cumulative number of species whose recorded elevation ranges intersected each interval, allowing for the identification of richness peaks in relation to the geometric centre of the domain.

The ecological niche breadth for each species was quantified as the proportion of the total elevation domain occupied, which was calculated only for the species with available data on elevation interval. This was calculated using the formula:

Proportional Niche Breadth = [(Max. Elevation – Min. Elevation) / Total Domain Range] × 100

Species were categorised based on their niche breadth to distinguish between elevation specialists (narrow ranges) and generalists (broad ranges). All calculations were performed using Microsoft Excel (version 2019).

## Results

### Family Conopidae Latreille, 1802

**Subfamily Conopinae Latreille, 1802**

**Tribe Brachyceraeini Zimina, 1960**

***Brachyceraea* Röder, 1893**

**1. *Brachyceraea brevicornis* (Loew, 1847)**

**Material examined**. Armenia – **Aragatsotn Prov**. • 1♂; Orgov; 40.3640°N, 44.2477°E; 1770 m; 06.08.2025; M. Aubert, V. Leclercq, R. Le Divelec leg. – **Kotayk Prov**. • 1♂; 09.08.1928; M. Makaryan leg. – **Yerevan** • 1♀; 16.06.1924; unknown collector – **Vayots Dzor Prov**. • 1♂, 1♀; Hermon; 39.8741°N, 45.4166°E; 1660 m; 28.07.2025; M. Aubert, V. Leclercq, R. Le Divelec leg. – **Syunik Prov**. • 1♂; Shvanidzor; 08.07.1929; A. Schelkovnikov leg. • 1♀; Vardanidzor; 38.9711°N, 46.2207°E; 1157 m; 19.06.2024; M. Aubert, V. Leclercq leg. • 2♂; Meghri; 38.9179°N, 46.2188°E; 812 m; 19.06.2024; M. Aubert, V. Leclercq leg.

**Published records**. Reported from Yerevan by Paramonov (1927).

**General distribution**. Greece, Southern European Russia, Syria, Lebanon, Turkey, Iran; Transcaucasia: Armenia (Chvála and Smith 1988; Stuke 2017).

**Habitats**. Mountain steppes and grasslands.

### Tribe Conopini Latreille, 1802

***Conops* Linnaeus, 1758**

**2. *Conops flavicauda* (Bigot, 1880)**

**Published records**. Reported from Armenia without locality data (Zimina 1976).

**General distribution**. East Mediterranean, Caucasus, Iran, Afghanistan, Tajikistan; recorded from Uganda in Afrotropical Region; Transcaucasia: Armenia (Chvála and Smith 1988; Stuke 2017).

**3. *Conops flavifrons* Meigen, 1824**

**Material examined**. Armenia – **Aragatsotn Prov**. • 1♀; Orgov; 40.3640°N, 44.2477°E; 1770 m; 06.08.2025; M. Aubert, V. Leclercq, R. Le Divelec leg. – **Ararat Prov**. • 2♀; Goravan; 39.8889°N, 44.7327°E; 950 m; 10.06.2024; M. Aubert, V. Leclercq, R. Le Divelec leg. • 2♀; Goravan; 39.8882°N, 44.7344°E; 963 m; 12.06.2025; N. Grigoryan leg. – **Yerevan** • 1♂, 3♀; Charents street; 40.1690°N, 44.5278°E; 1050 m; 26.07.2025; M. Aubert, V. Leclercq, R. Le Divelec leg. – **Syunik Prov**. • 1♀; Zangezur [corresponds to southern part of Syunik province]; 24.07.1924; Izmailov leg. • 1♀; Alvank; 38.9176°N, 46.3380°EE; 480 m; 20.07.2024; light traps.

**Published records**. As *C. kroeberi*Paramonov 1927 described from Yerevan together with *C. k.* var. *immaculatus*Paramonov 1927 (Paramonov 1927); Zimina (1976) reported the species from South Armenia without exact locality data.

**General distribution**. North Africa; from Western Europe to Southern European Russia, Turkey, Kazakhstan, and Central Asia; Yemen; ?China; Transcaucasia: Armenia (Chvála and Smith 1988; Stuke 2017).

**Habitats**. Mountain steppes and grasslands.

**4. *Conops silaceus* Wiedemann in Meigen, 1824**

**Material examined**. Armenia – **Aragatsotn Prov**. • 1♀; Orgov; 40.3640°N, 44.2477°E; 1770 m; 06.08.2025; M. Aubert, V. Leclercq, R. Le Divelec leg.

**Published records**. Armenia without locality data (Chvála and Smith 1988; Zimina 2000)

**General distribution**. Western Palearctic region from Iberian Peninsula to Southern European Russia, Turkey, Western Kazakhstan; Transcaucasia: Armenia (Chvála and Smith 1988; Stuke 2017).

**Habitats**. Mountain steppes and grasslands.

### *Leopoldius* Rondani, 1843

**5. *Leopoldius coronatus* (Rondani, 1857)**

**Material examined**. Armenia – **Syunik Prov**. • 1♂, 1♀; Shikahogh State Reserve; 39.0902°N, 46.4783°E; 1013 m; 15.07.2025, 11.08.2024; N. Grigoryan leg.

**Published records**. Reported from Kirovakan (currently Vanadzor, Lori Province) (Zimina 1976).

**General distribution**. Central and Southern Europe, southern European Russia, Turkey, North Africa; Transcaucasia: Armenia (Chvála and Smith 1988; Stuke 2017).

**Habitats**. Forested landscapes along edges.

### Tribe Physocephalini Smith & Peterson, 1987

***Physocephala* Schiner, 1861**

6. *Physocephala antiqua (Wiedemann, 1830)

Figs 9, 10

**Material examined**. Armenia – **Ararat Prov**. • 1♀; Eghegnut; 40.0796°N, 44.1317°E; 870 m; 27.07.2025; M. Aubert, V. Leclercq, R. Le Divelec leg.

**Published records**. First record for Armenia.

**General distribution**. North Africa; Eastern Mediterranean, West and Central Asia, Southern European Russia to Western Siberia, Mongolia, and China (Chvála and Smith 1988; Stuke 2017).

**Habitats**. A single specimen was collected in a wetland within the semi-desert landscape belt, which does not allow reliable identification of the species’ typical habitat.

**7. *Physocephala chrysorrhoea* (Meigen, 1824)**

**Material examined**. Armenia – **Armavir Prov**. • 1♂, 1♀; Zartonk; 40.0994°N, 44.1298°E, 870 m; 27.07.2025; M. Aubert, V. Leclercq, R. Le Divelec leg. – **Vayots Dzor Prov**. • 1♂; Getap; 40.3747°N, 43.6183°E; 1316 m; 03.07.2024; N. Grigoryan leg.

**Published records**. Reported from Yerevan (Paramonov 1927).

**General distribution**. North Africa; Europe from Belgium and Spain to Greece, Southern European Russia, Kazakhstan, Western and Central Asia, Siberia, Mongolia, China; Transcaucasia: Armenia (Chvála and Smith 1988; Stuke 2017).

**Habitats**. From semi-desert landscapes to mountain steppes and grasslands.

**8. *Physocephala nigra* (De Geer, 1776)**

**Material examined**. Armenia – **Kotayk Prov**. • 1♂; Hankavan; 40.6222°N, 44.4663°E; 2046 m; 04.07.2025; N. Grigoryan leg.

**Published records**. Reported without locality data (Zimina 1976).

**General distribution**. North Africa; Europe from Great Britain and Fennoscandia to Southern European Russia, Kazakhstan, Central Asia, Mongolia, China, and Russian Far East; Transcaucasia: Armenia, Azerbaijan, Georgia (Chvála and Smith 1988; Stuke 2017).

**Habitats**. The only specimen was collected in a subalpine meadow with abundant flowering plants.

**9. *Physocephala pusilla* (Meigen, 1824)**

**Material examined**. Armenia – **Aragatsotn Prov**. • 1♀; Agarak; 40.2952°N, 44.2786°E, 1070 m; 06.08.2025; M. Aubert, V. Leclercq, R. Le Divelec leg. – **Ararat Prov**. • 1♂; Azat Reservoir; 40.0761°N, 44.6276°E; 1100 m; 21.07.2025; M. Kalashian leg. • 1♀; Eghegnut; 40.0796°N, 44.1317°E; 870 m; 27.07.2025; M. Aubert, V. Leclercq, R. Le Divelec leg. – **Armavir Prov**. • 1♂, 1♀; Shahumyan; 40.2064°N, 44.3225°E; 940 m; 07.08.2025; M. Aubert, V. Leclercq, R. Le Divelec leg. • 1♂; Amberd; 40.2489°N, 44.2771°E; 950 m; 07.08.2025; M. Aubert, V. Leclercq, R. Le Divelec leg. – **Yerevan** • 1♀; 25.06.1924; A. Schelkovnikov leg. • 1♂; Victory Park; 40.195°N, 44.517°E; 1125 m; 09.06.2024; M. Aubert, V. Leclercq leg. • 5♂; Charents street; 40.1690°N, 44.5278°E; 1050 m; 26.07.2025; M. Aubert, V. Leclercq, R. Le Divelec leg. –**Vayots Dzor Prov**. • 1♂; Getap; 40.3747°N, 43.6183°E; 1316 m; 03.07.2024; N. Grigoryan leg. – **Syunik Prov**. • 1♀; Megri; 12.06.1925; M. Rjabov leg. • 3♂; Megri; 38.9179°N, 46.2188°E; 812 m; 19.06.2024; M. Aubert, V. Leclercq leg. • 1♂; Arevik National Park “Boghaqar”; 38.9892°N, 46.1865°E; 1342 m; 28.08.2025; T. Ghrejian leg.

**Published records**. Reported from Yerevan (Paramonov 1927).

**General distribution**. North Africa; from Southern and Central Europe through Southern European Russia to Kazakhstan, Southern Siberia, China, Mongolia; Western Asia; Transcaucasia: Armenia (Kröber 1936; Chvála and Smith 1988; Stuke 2017).

**Habitats**. From lowland semi-desert landscapes to mountain steppes.

**10. *Physocephala rufipes* (Fabricius, 1781)**

**Material examined**. Armenia – **Aragatsotn Prov**. • 1♀; Orgov; 40.3640°N, 44.2477°E; 1770 m; 06.08.2025; M. Aubert, V. Leclercq, R. Le Divelec leg. – **Vayots Dzor Prov**. • 1♂, 1♀; Hermon; 39.8741°N, 45.4166°E; 1660 m; 28.07.2025; M. Aubert, V. Leclercq, R. Le Divelec leg.

**Published records**. First explicit report for Armenia.

**General distribution**. North Africa; from British Islands to Turkey, European Russia, Siberia, Kazakhstan, Central Asia, Mongolia, and China; Transcaucasia (Chvála and Smith 1988; Stuke 2017).

**Habitats**. Mountain steppes to subalpine meadows.

**11. *Physocephala variegata* (Meigen, 1824)**

**Material examined**. Armenia – **Ararat Prov**. • 1♀; Goravan; 39.8882°N, 44.7344°E; 963 m; 12.06.2025; N. Grigoryan leg.

**Published records**. First explicit report for Armenia.

**General distribution**. Southern Europe to Southern European Russia, Egypt, Turkey, Iran, Afghanistan, Turkmenistan, Mongolia, and China; Transcaucasia (Chvála and Smith 1988).

**Habitats**. The only specimen was collected in a semi-desert landscape.

**12. *Physocephala vittata* (Fabricius, 1794)**

**Material examined**. Armenia – **Syunik Prov**. • 1♂; Vardanidzor; 38.9711°N, 46.2207°E; 1157 m; 19.06.2024; M. Aubert, V. Leclercq leg.

**Published records**. As *P.v.* var. *abdominalis* Kröber, 1915 reported from Yerevan (Kröber 1915a; Paramonov 1927).

**General distribution**. North Africa; from Scandinavia to Southern and Eastern Europe, Central and Southern European Russia, Western and Central Asia, Kazakhstan, Mongolia, China; Afrotropical Region; Transcaucasia: Armenia (Chvála and Smith 1988; Stuke 2017).

**Habitats**. A single specimen was collected at the edge of a sparse juniper forest.

### Tribe Tropidomyiini Zimina, 1960

***Tropidomyia* Williston, 1888**

**13. *Tropidomyia aureifacies* Krober, 1915**

**Published records**. Reported by Zimina (1976) from Southern Armenia.

**General distribution**. Greece, Turkey, Syria, Afghanistan, Kazakhstan, Central Asia, China, Japan; Transcaucasia: Armenia, Azerbaijan (Chvála and Smith 1988; Stuke 2017).

### Subfamily Dalmanniinae Hendel, 1916

***Dalmannia* Robineau-Desvoidy, 1830**

**14. *Dalmannia aculeata* (Linnaeus, 1761)**

**Published records**. Reported from Yerevan by Paramonov (1927) (as *D. a.* var. *nigrifemorata*Paramonov 1927).

**General distribution**. North Africa; Western Europe to Southern European Russia, Turkey, Iran, Central Asia; Transcaucasia: Armenia (Chvála and Smith 1988; Stuke 2017).

**15. *Dalmannia marginata* (Meigen, 1824)**

**Published records**. Reported by Zimina (1976) from Southern Armenia.

**General distribution**. Europe from Fennoscandia to Southern Europe and Southern European Russia, Western Asia, Turkmenistan; Transcaucasia: Armenia (Chvála and Smith 1988; Stuke 2017).

**16. *Dalmannia punctata* (Fabricius, 1794)**

**Material examined**. Armenia – **Aragatsotn Prov**. • 1♂, Garnahovit; 40.4996°N, 43.9739°E; 2260 m; 02.05.2025; M. Kalashian leg. – **Ararat Prov**. • 1♂; Lanjar; 39.8381°N, 44.9706°E; 1975 m; 14.06.2024; M. Aubert, V. Leclercq leg.

**Published records**. Reported from Yerevan by Paramonov (1927).

**General distribution**. From Western Europe and Fennoscandia to European Russia, Turkey, Iran and Central Asia; Transcaucasia: Armenia (Chvála and Smith 1988; Stuke 2017).

**Habitats**. Montane meadows.

### Subfamily Myopinae Macquart, 1834

**Tribe Myopini Macquart, 1834**

***Merziella* Stuke, 2014**

**17. *Merziella longirostris* (Lyneborg, 1962)**

**Material examined**. Armenia – **Vayots Dzor Prov**. • 1♂, 1♀; Vardahovit; 39.9750°N, 45.5496°E; 2560 m; 30.07.2025; M. Aubert, V. Leclercq, R. Le Divelec leg.

**Published records**. Reported from “Zangezur” (approximately corresponds to southern part of modern Syunik Province) (Zimina 1976, as *Thecophora*).

**General distribution**. Central and Southern Europe, Belarus, Ukraine, Western Siberia, Kazakhstan, Central Asia, Turkey; Transcaucasia: Armenia (Chvála and Smith 1988, as *Thecophora*; Stuke 2017).

**Habitats**. Montane meadows.

### *Myopa* Fabricius, 1775

18. *Myopa buccata (Linnaeus, 1758)

Figs 3–5

**Figures 1–8.**
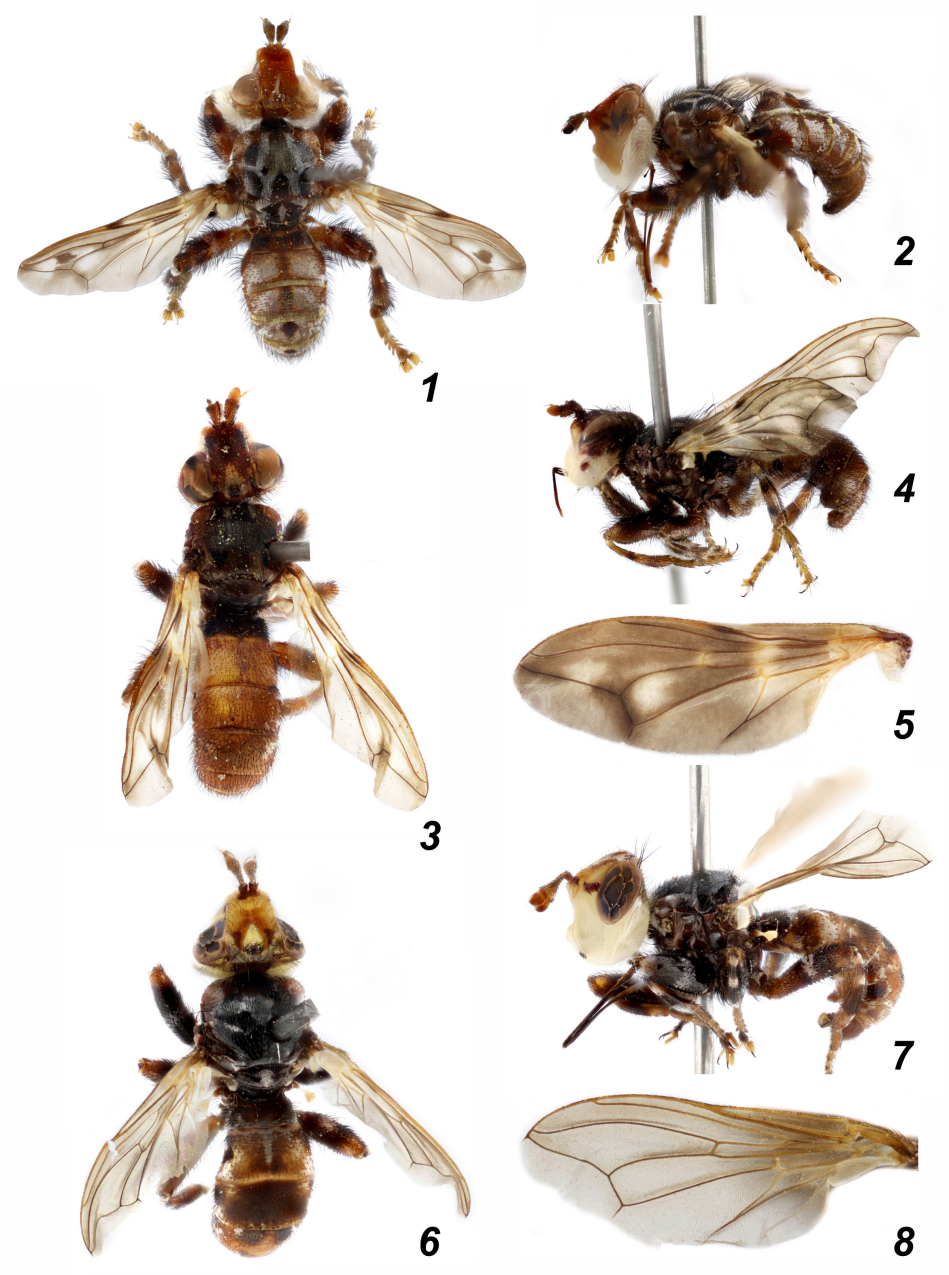
*Myopa* spp. **1, 2**. *M. picta* Panzer, 1797, Gorayk; **3–5**. *M. buccata* (Linnaeus, 1758), Vardenik; **6–8**. *M. variegata* Meigen, 1804, Nshkhark. Habitus dorsally (1, 3, 6), habitus laterally (2, 4, 7), wing (5, 8).

**Material examined**. Armenia – **Gegharkunik Prov**. • 1♂; Vardenik; 40.1169°N, 45.4571°E; 2069 m; 13.06.2024; M. Aubert, V. Leclercq leg.

**Published records**. First record for Armenia.

**General distribution**. Palaearctic Region from Iberian Peninsula to Japan (Chvála and Smith 1988; Stuke 2017).

**Habitats**. The only specimen was collected in a montane meadow.

**19. *Myopa dorsalis* Fabricius, 1794**

**Material examined**. Armenia – **Shirak Prov**. • 1♀; Gyumri; 27.06.1964; Kh. M. Arutyunyan leg. – **Lori Prov**. • 1♂; Stepanavan; 28.06.1920; unknown collector – **Ararat Prov**. • 1♀; Lanjar; 39.8381°N, 44.9706°E; 1975 m; 14.06.2024; M. Aubert, V. Leclercq leg.

**Published records**. Reported by Paramonov (1927) from Stepanavan (Lori Province).

**General distribution**. North Africa; Europe from Spain and Fennoscandia to European Russia, Turkey, Syria, Iran, Turkmenistan, Kazakhstan, China; India; Transcaucasia: Armenia (Chvála and Smith 1988; Stuke 2017).

**Habitats**. Montane meadows.

**20. *Myopa morio* Meigen, 1804**

**Published records**. Southern Armenia without exact locality data (Zimina 1963a, 1976).

**General distribution**. Central and Southern Europe to Central and Southern European Russia, Turkey, Iran, Kyrgyzstan; Transcaucasia: Armenia (Chvála and Smith 1988; Stuke 2017).

**21. *Myopa pallida* Krôber, 1916**

**Material examined**. Armenia – **Gegharkunik Prov**. • 1♂; Vardenik; 40.1169°N, 45.4571°E; 2069 m; 13.06.2024; M. Aubert, V. Leclercq leg.

**Published records**. Reported from Dilijan (Tavush Province) (Zimina 1963a).

**General distribution**. Turkey; all three Transcaucasian countries (Chvála and Smith 1988; Stuke 2017).

**Habitats**. The only specimen was collected in a montane meadow.

22. *Myopa picta Panzer, 1797

Figs 1, 2

**Material examined**. Armenia – **Syunik Prov**. • 1♂; Gorayk; 39.6760°N, 45.7325°E; 2205 m; 17.06.2024; M. Aubert, V. Leclercq leg.

**Published records**. First record for Armenia.

**General distribution**. North Africa; from Western Europe to European Russia, Western and Central Asia, China; India; Ethiopia; Transcaucasia: Azerbaijan (Chvála and Smith 1988; Stuke 2017).

**Habitats**. The only specimen was collected in the montane meadow.

**23. *Myopa testacea* (Linnaeus, 1767)**

**Material examined**. Armenia – **Gegharkunik Prov**.: 2♂, env. Vardenik, 40.1169°N, 45.4571°E, 2069 m, 13.06.2024, M. Aubert, V. Leclercq leg. – **Yerevan**: 1♀, 1♂, 22.05.1924, 28.05.1924, A. Schelkovnikov leg. – **Vayots Dzor Prov**.: 2♂, env. Nshkhark, 39.9422°N, 45.2356°E, 2138 m, 14.06.2024, M. Aubert, V. Leclercq leg.

**Published records**. Armenia without locality data (Kröber 1936).

**General distribution**. Palearctic Region from Great Britain and the Pyrenees to Japan; India; Transcaucasia: Armenia (Chvála and Smith 1988; Stuke 2017).

**Habitats**. Montane meadows.

24. *Myopa variegata Meigen, 1804

Figs 6–8

**Material examined**. Armenia – **Aragatsotn Prov**. • 2♂; Orgov; 40.36404°N, 44.2477°E; 1770 m; 06.08.2025; M. Aubert, V. Leclercq, R. Le Divelec leg. – **Vayots Dzor Prov**. • 1♂; Nshkhark; 39.97035°N, 45.2437°E; 2310 m; 01.08.2025; M. Aubert, V. Leclercq, R. Le Divelec leg.

**Published records**. First record for Armenia.

**General distribution**. From Western Europe to European Russia, Turkey, Kazakhstan, Southern Siberia, Mongolia, China, and Russian Far East; Saudi Arabia (Chvála and Smith 1988; Stuke 2017).

**Habitats**. Montane meadows.

### *Myopotta* Zimina, 1969

**25. *Myopotta pallipes* (Wiedemann, 1824)**

**Material examined**. Armenia – **Ararat Prov**. • 1♂; Lanjar; 39.8381°N, 44.9706°E; 1975 m; 14.06.2024; M. Aubert, V. Leclercq leg. – **Vayots Dzor Prov**. • 1♀; Nshkhark; 39.9422°N, 45.2356°E; 2138 m; 14.06.2024; M. Aubert, V. Leclercq leg.

**Published records**. Reported from Yerevan by Kröber (1915b, as *Melanosoma*).

**General distribution**. From Central and Southern Europe and Finland to Northern and Central European Russia, Turkey, Iran, Western Siberia, Kazakhstan, Kyrgyzstan, Mongolia, China (Chvála and Smith 1988; Stuke 2017).

**Habitats**. Montane meadows.

### Tribe Thecophorini Gibson in Gibson and Skevington 2013

***Thecophora* Rondani, 1845**

26. *Thecophora atra (Fabricius, 1775)

Figs 11, 15

**Figures 9–16.**
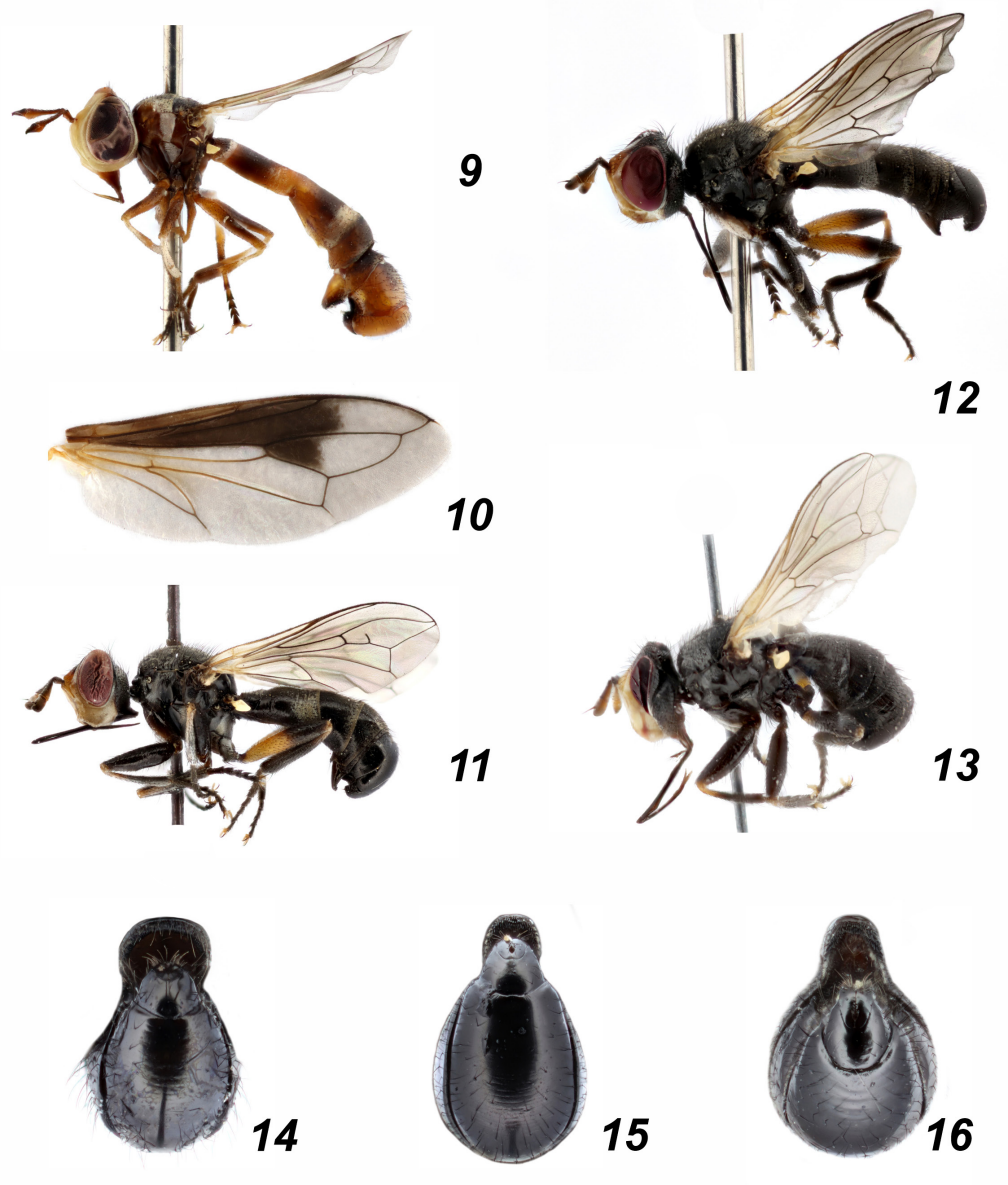
*Physocephala antiqua* (Wiedemann, 1830) and *Thecophora* spp. **9, 10**. *Ph. antiqua*, Eghegnut; **11, 15**. *Th. atra* (Fabricius, 1775), Shikahogh State Reserve; **12, 16**. *Th. fulvipes* (Robineau-Desvoidy, 1830), Papanino; **13, 14**. *Th. cinerascens* (Meigen, 1804), Lichk. Habitus laterally (9, 11, 12, 13), wing (10), theca (14, 15, 16).

**Figure 17.**
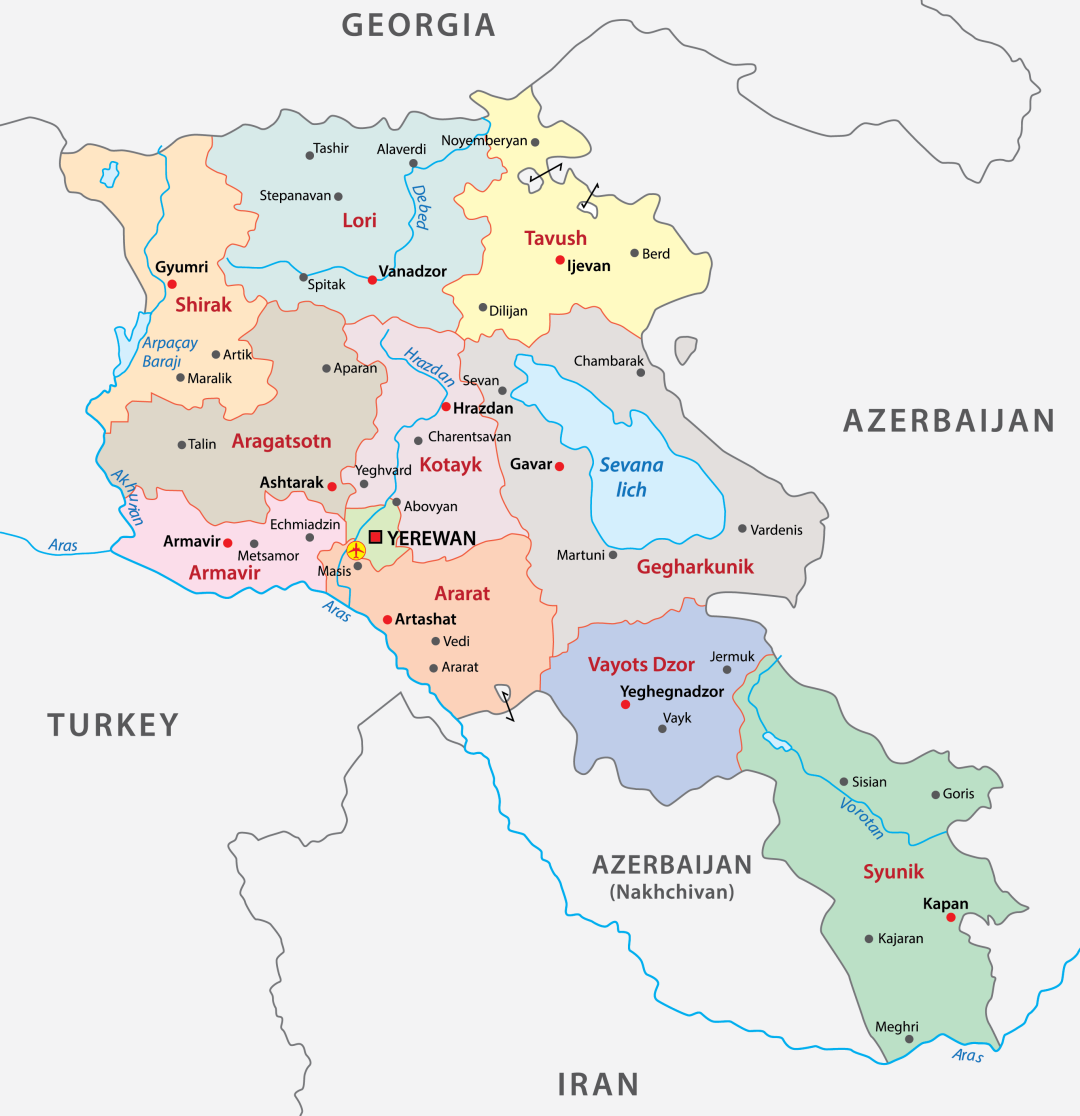
Provinces of Armenia (© Worldatlas contributors).

**Material examined**. Armenia – **Shirak Prov**. • 1♂; Tavshut; 41.0761°N, 43.8047°E; 2010 m; 19.08.2025; M. Kalashian leg. – **Tavush Prov**. • 1♂; Haghartsin; 40.8235°N, 44.9570°E; 2000 m; 03.08.2025; M. Aubert, V. Leclercq, R. Le Divelec leg. – **Aragatsotn Prov**. • 1♂; Orgov; 40.3640°N, 44.2477°E; 1770 m; 06.08.2025; M. Aubert, V. Leclercq, R. Le Divelec leg. – **Vayots Dzor Prov**. • 1♀; Vardahovit; 39.9585°N, 45.5529°E; 2400 m; 29.07.2025; M. Aubert, V. Leclercq, R. Le Divelec leg.; • 1♂; Nshkhark; 39.9703°N, 45.2437°E; 2310 m; 01.08.2025; M. Aubert, V. Leclercq, R. Le Divelec leg. – **Syunik Prov**. • 1♀; Gorayk; 39.6760°N, 45.7325°E; 2205 m; 17.06.2024; M. Aubert, V. Leclercq leg.; • 1♂; Meghri; 38.9179°N, 46.2188°E; 812 m; 19.06.2024; M. Aubert, V. Leclercq leg.; • 1♂; Shishkert; 39.0404°N, 46.3929°E; 1700 m; 27.08.2025; on *Chondrilla* sp.; M. Kalashian leg.; • 1♀; Shikahogh State Reserve; 38.9960°N, 46.3776°E; 1529 m; 27.08.2025; T. Ghrejian leg.

**Published records**. First record for Armenia.

**General distribution**. Palaearctic Region from Great Britain to Japan; India, Sri Lanka (Chvála and Smith 1988; Stuke 2017). According to Stuke (2017) records from Asian countries need verification.

**Habitats**. The species primarily inhabits montane meadows but also occurs along the edges in forested areas.

27. *Thecophora cinerascens (Meigen, 1804)

Figs 13, 14

**Material examined**. Armenia – **Gegharkunik Prov**. • 2♂; Tsapatagh; 40.3966°N, 45.4933°E; 2037 m; 12.06.2024; M. Aubert, V. Leclercq leg. • 1♀; Selim Pass; 39.9421°N, 45.2378°E; 2140 m; 01.08.2025; M. Aubert, V. Leclercq, R. Le Divelec leg. – **Aragatsotn Prov**. • 1♂; Orgov; 40.3640°N, 44.2477°E; 1770 m; 06.08.2025; M. Aubert, V. Leclercq, R. Le Divelec leg. Armenia – **Vayots Dzor Prov**. • 1♂; Vardahovit; 39.9585°N, 45.5529°E; 2400 m; 29.07.2025; M. Aubert, V. Leclercq, R. Le Divelec leg. • 4♂; Arates; 39.9084°N, 45.4303°E; 2100 m; 31.07.2025; M. Aubert, V. Leclercq, R. Le Divelec leg. • 1♀; Nshkhark; 39.9703°N, 45.2437°E; 2310 m; 01.08.2025; M. Aubert, V. Leclercq, R. Le Divelec leg. – **Syunik Prov**. • 1♂, 2♀; Aghvani; 39.3629°N, 46.2998°E; 1832 m; 20.06.2024; M. Aubert, V. Leclercq leg. • 1♀; Lichk; 39.0992°N, 46.1632°E; 2080 m; 28.08.2025; M. Kalashian leg.

**Published records**. First record for Armenia.

**General distribution**. Great Britain, Central and Southern Europe, Sweden, European Russia, Turkey, Iran, Kyrgyzstan; Transcaucasia: Azerbaijan (Stuke 2017).

**Habitats**. Mountain steppes and meadows, sometimes also forest edges.

**28. *Thecophora distincta* (Meigen & Wiedemann, 1820)**

**Material examined**. Armenia – **Vayots Dzor Prov**. • 2♂; Nshkhark; 39.9703°N, 45.2437°E; 2310 m; 01.08.2025; M. Aubert, V. Leclercq, R. Le Divelec leg.

**Published records**. Reported from Yerevan by Paramonov (1927, as *Occemyia*).

**General distribution**. From Western Europe and Fennoscandia to European part of Russia east to Kazakhstan, Kyrgyzstan, Mongolia, China and Russian Far East; Tunisia, Turkey, Iran, Turkmenistan; Ethiopia; Transcaucasia: Armenia (Kröber 1936; Chvála and Smith 1988; Stuke 2017).

**Habitats**. Collected in the mountain meadow.

**29. *Thecophora fulvipes* (Robineau-Desvoidy, 1830)**

Figs 12, 16

**Material examined**. Armenia – **Shirak Prov**. • 2♂; Tavshut; 41.0761°N, 43.8047°E; 2010 m; 19.08.2025; M. Kalashian leg. – **Tavush Prov**. • 1♀; Papanino; 40.7848°N, 44.8431°E; 1650 m; 02.08.2025; M. Aubert, V. Leclercq, R. Le Divelec leg. • 1♂; Gosh; 40.7580°N, 45.0219°E; 930 m; 09.08.2025; N. Grigoryan leg. – **Vayots Dzor Prov**. • 1♀; Arates; 39.9084°N, 45.4303°E; 2100 m; 31.07.2025; M. Aubert, V. Leclercq, R. Le Divelec leg.

**Published records**. Reported from Armenia without locality data (Zimina 1976; Chvála and Smith 1988).

**General distribution**. North Africa; Europe from Great Britain and Fennoscandia to European Russia east to Mongolia, China and Russian Far East; Turkey, Iran; Transcaucasia: Armenia, Georgia, Azerbaijan (Zimina 1976; Chvála and Smith 1988; Stuke 2017).

**Habitats**. Mountain steppes and meadows, sometimes also forest edges.

### Subfamily Sicinae Zimina, 1960

***Sicus* Scopoli, 1763**

**30. *Sicus ferrugineus* (Linnaeus, 1761)**

**Material examined**. Armenia – **Lori Prov**. • 2♀; Stepanavan; 07.08.1920; unknown collector – **Tavush Prov**. • 1♂; Papanino; 40.7848°N, 44. 8431°E; 1650 m; 02.08.2025; M. Aubert, V. Leclercq, R. Le Divelec leg. • 1♂; Haghartsin; 40.8235°N, 44.9570°E; 2000 m; 03.08.2025; M. Aubert, V. Leclercq, R. Le Divelec leg. – **Aragatsotn Prov**. • 2♂; Hartavan; 01.07.1920; unknown collector – **Vayots Dzor Prov**. • 1♂; Nshkhark; 39.9703°N, 45.2437°E; 2310 m; 01.08.2025; M. Aubert, V. Leclercq, R. Le Divelec leg. – **Syunik Prov**. • 2♂; Lichk; 39.0984°N, 46.1637°E; 2048 m; 25.07.2024; N. Grigoryan leg. • 1♂; Lichk; 39.0991°N, 46.1631°E; 2077 m; 16.07.2025; M. Kalashian leg.

**Published records**. Reported from Lori (Vanadzor and Stepanavan) and Kotayk (Mt. Arailer, Hankavan) Provinces (Paramonov 1927).

**General distribution**. Palaearctic Region from Great Britain and the Pyrenees to Japan and Korea; India; Transcaucasia: Armenia (Chvála and Smith 1988; Stuke 2017).

**Habitats**. Mountain steppes and meadows, sometimes also forest edges.

**31. *Sicus nigritarsis* Zimina, 1975**

**Material examined**. Armenia – **Tavush Prov**. • 2♂; Haghartsin; 40.8235°N, 44.9570°E; 2000 m; 03.08.2025; M. Aubert, V. Leclercq, R. Le Divelec leg.

**Published records**. Among the paratypes of the species some originated from Kotayk Province (Hankavan and Arzakan) (Zimina 1975).

**General distribution**. Italy, Balkans, Turkey, Central and South European Russia to Western Siberia; Transcaucasia: Armenia (Chvála and Smith 1988; Stuke 2017).

**Habitats**. Collected in forested landscape near edge.

**Subfamily Zodioninae Rondani, 1856**

### *Zodion* Latreille, 1797

**32. *Zodion cinereum* (Fabricius, 1794)**

**Material examined**. Armenia – **Tavush Prov**. • 1♀; Gosh; 40.7580°N, 45.0219°E; 930 m; 09.08.2025; N. Grigoryan leg. – **Gegharkunik Prov**. • 1♂; Tsapatagh; 15.07.1928; A. Schelkovnikov leg. – **Aragatsotn Prov**. • 4♂, 1♀; Orgov; 40.3640°N, 44.2477°E; 1770 m; 06.08.2025; M. Aubert, V. Leclercq, R. Le Divelec leg. – **Ararat Prov**. • 1♀; Armash; 10.06.1930; A. Schelkovnikov leg. – **Vayots Dzor Prov**. • 1♂; Vardahovit; 39.9784°N, 45.5470°E; 2630 m; 30.07.2025; M. Aubert, V. Leclercq, R. Le Divelec leg. • 2♂; Arates; 39.9084°N, 45.4303°E; 2100 m; 31.07.2025; M. Aubert, V. Leclercq, R. Le Divelec leg.

**Published records**. Reported from Yerevan (Kröber 1915c; Paramonov 1927).

**General distribution**. Palaearctic Region from Great Britain to Japan; Subsaharan Africa; India; Transcaucasia: Armenia (Chvála and Smith 1988; Stuke 2017).

**Habitats**. Mountain meadows, sometimes also forest edges.

## Discussion

The present study provides the first comprehensive, country-level checklist of Conopidae (Diptera) for Armenia, documenting 32 species from 12 genera, including six species newly recorded for the country. In total, 126 specimens were collected; noticeable predominance of males over females was observed (81 males and 45 females with ratio of 1.8:1). A comparison of species richness in neighbouring countries—based on data from the World Catalogue of Insects (Stuke 2017) and the comprehensive work on the Diptera of Turkey (Koçak and Kemal 2013)—indicates 53 species reported from Turkey and 29 from Iran. Currently, very limited data are available for Georgia and Azerbaijan, where 12 and seven species have been reported, respectively (Stuke and Kehlmaier 2008; Stuke 2017; Tarkhnishvili et al. 2026). Furthermore, according to the Georgian Biodiversity Database, several species recorded from Georgia require confirmation. Given the larger land area and greater ecological heterogeneity of these neighbouring countries, the conopid diversity reported here for Armenia appears relatively high. This pattern likely reflects historical under-sampling in adjacent regions rather than genuinely lower species richness. The observed disparity underscores the need for intensified faunistic surveys across the Transcaucasia to clarify the true distribution patterns of Conopidae both in Armenia and the region.

Habitat preferences of each species or genus were assessed based solely on the available distribution data. The elevation gradient (domain) spanned from 480 m to 2630 m, and the Conopidae fauna of Armenia showed clear habitat associations along this gradient. Analysis of species distributions revealed a pattern consistent with the mid-domain effect (MDE), as maximum species richness was observed between 1000 m and 1800 m, coinciding with the geometric midpoint of the domain.

Most species were recorded in alpine and subalpine meadows (≈ 2000–2600 m) which were predominantly occupied by numerous taxa such as *Dalmannia punctata*, *Merziella longirostris*, *Myopotta pallipes*, *Myopa* spp., *Physocephala nigra*, and *Thecophora distincta*, indicating a specialised affinity for high-mountain environments with abundant flowering plants and Hymenopteran hosts. Several species, such as *Brachyceraea brevicornis*, *Conops* spp., and certain *Physocephala* species, were found in mountain steppes and meadows (≈ 800–2000 m). These findings highlight the importance of open, flower-rich grasslands for adult feeding and larval development. Some species from these landscapes, including *Zodion cinereum*, *Thecophora cinerascens*, and *T. fulvipes*, were also found in forested areas. *Leopoldius coronatus* can be considered a typical inhabitant of forested landscapes, while only three species of *Physocephala* were observed in the semi-desert belt.

Quantitatively, individual species showed varying niche breadths as a proportion of the total domain. The average proportional niche breadth across the observed species was 35.33% (SD = 23.27). This relatively high standard deviation reflects a high degree of variation in range sizes, encompassing both narrow-range specialists and broad-range generalists. The ecological niches ranged from highly specialised, such as *Physocephala rufipes* (occupying only 5.1% of the domain), to broadly distributed, such as *Zodion cinereum* (occupying 79.1% of the domain). Consequently, while species like *Z. cinereum* appear to be elevation generalists with broad ecological amplitude, the narrow ranges of the *Myopotta* and *Dalmannia* genera at higher elevations likely indicate specialised adaptations to alpine conditions.

Therefore, mid-elevation mountain steppes and high-elevation alpine meadows, characterised by flower-rich open habitats, constitute the primary ecological niches for quite diverse and relatively stenoecious conopid communities, while forest edges and lowland flower-rich areas provide secondary habitats for more generalist species and only a few stenoecious species.

The diversity documented in this study provides a foundation for investigating the broader ecological roles of conopid flies as obligate endoparasitoids of bees and wasps. Despite their ecological importance, host associations of many conopid species remain poorly understood, especially in the Caucasus region. Further targeted field surveys combined with rearing studies are therefore essential to clarify species-specific host–parasitoid relationships within the Armenian fauna. Such integrative approaches would not only improve the taxonomic completeness and accuracy of the national checklist but also provide valuable insights into pollinator–parasitoid dynamics, which are increasingly recognised as an important component of ecosystem functioning and conservation planning.

## Additional information

### Artificial Intelligence (AI) use

The authors accept full responsibility for the content of the manuscript, including the disclosure of any use of AI.

No AI tools were used in the preparation of this manuscript.

### Author ORCIDs

N. Grigoryan https://orcid.org/0000-0001-8079-8574

M. Aubert https://orcid.org/0000-0003-2713-9371

G. Karagyan https://orcid.org/0000-0001-7708-2145

M. Kalashian https://orcid.org/0000-0002-2448-9547

T. Ghrejyan https://orcid.org/0000-0003-4359-2870

### Data availability

All of the data that support the findings of this study are available in the main text or Supplementary Information.

## Supplementary materials

Supplementary material 1 Conopidae distribution data Data type xlsx Explanation note The supplementary file provides comprehensive data on species distribution, including all examined locality records and associated occurrence information, and are available under open access. https://binary.pensoft.net/file/1723951This dataset is made available under the Open Database License (http://opendatacommons.org/licenses/odbl/1.0/). The Open Database License (ODbL) is a license agreement intended to allow users to freely share, modify, and use this Dataset while maintaining this same freedom for others, provided that the original source and author(s) are credited. Nonna Grigoryan, Matthieu Aubert, Gayane Karagyan, Mark Kalashian, Tigran Ghrejyan
